# Worldwide Protein Data Bank biocuration supporting open access to high-quality 3D structural biology data

**DOI:** 10.1093/database/bay002

**Published:** 2018-02-07

**Authors:** Jasmine Y Young, John D Westbrook, Zukang Feng, Ezra Peisach, Irina Persikova, Raul Sala, Sanchayita Sen, John M Berrisford, G Jawahar Swaminathan, Thomas J Oldfield, Aleksandras Gutmanas, Reiko Igarashi, David R Armstrong, Kumaran Baskaran, Li Chen, Minyu Chen, Alice R Clark, Luigi Di Costanzo, Dimitris Dimitropoulos, Guanghua Gao, Sutapa Ghosh, Swanand Gore, Vladimir Guranovic, Pieter M S Hendrickx, Brian P Hudson, Yasuyo Ikegawa, Yumiko Kengaku, Catherine L Lawson, Yuhe Liang, Lora Mak, Abhik Mukhopadhyay, Buvaneswari Narayanan, Kayoko Nishiyama, Ardan Patwardhan, Gaurav Sahni, Eduardo Sanz-García, Junko Sato, Monica R Sekharan, Chenghua Shao, Oliver S Smart, Lihua Tan, Glen van Ginkel, Huanwang Yang, Marina A Zhuravleva, John L Markley, Haruki Nakamura, Genji Kurisu, Gerard J Kleywegt, Sameer Velankar, Helen M Berman, Stephen K Burley

**Affiliations:** 1 RCSB Protein Data Bank, Center for Integrative Proteomics Research, Rutgers, The State University of New Jersey, 174 Frelinghuysen Road, Piscataway, NJ 08854, USA; 2 Protein Data Bank in Europe (PDBe), European Molecular Biology Laboratory, European Bioinformatics Institute (EMBL-EBI), Wellcome Genome Campus, Hinxton, Cambridgeshire CB10 1SD, UK; 3 PDBj, Institute for Protein Research, Osaka University, 3-2 Yamadaoka, Suita-shi, Osaka 565-0871, Japan; 4 BMRB, BioMagResBank, University of Wisconsin-Madison, 433 Babcock Drive, Madison, WI 53706, USA; 5 RCSB Protein Data Bank, San Diego Supercomputer Center and Skaggs School of Pharmacy and Pharmaceutical Sciences, University of California San Diego, 9500 Gilman Dr., La Jolla, CA 92093, USA; 6 Institute for Quantitative Biomedicine, Rutgers, The State University of New Jersey, 174 Frelinghuysen Road, Piscataway, NJ 08854, USA; 7 Rutgers Cancer Institute of New Jersey, Rutgers, The State University of New Jersey, Little Albany St, New Brunswick, NJ 08901, USA

## Abstract

The Protein Data Bank (PDB) is the single global repository for experimentally determined 3D structures of biological macromolecules and their complexes with ligands. The worldwide PDB (wwPDB) is the international collaboration that manages the PDB archive according to the *FAIR* principles: Findability, Accessibility, Interoperability and Reusability. The wwPDB recently developed OneDep, a unified tool for deposition, validation and biocuration of structures of biological macromolecules. All data deposited to the PDB undergo critical review by wwPDB Biocurators. This article outlines the importance of biocuration for structural biology data deposited to the PDB and describes wwPDB biocuration processes and the role of expert Biocurators in sustaining a high-quality archive. Structural data submitted to the PDB are examined for self-consistency, standardized using controlled vocabularies, cross-referenced with other biological data resources and validated for scientific/technical accuracy. We illustrate how biocuration is integral to PDB data archiving, as it facilitates accurate, consistent and comprehensive representation of biological structure data, allowing efficient and effective usage by research scientists, educators, students and the curious public worldwide.

**Database URL**: https://www.wwpdb.org/

## Introduction

The Protein Data Bank (1) (PDB, pdb.org) was established in 1971 with just seven X-ray crystal structures and was the first open-access digital biological data resource. Today, the PDB is the single global archive for 3D macromolecular structure data, containing >130 000 structures determined by macromolecular crystallography (MX; using X-ray photons, electrons or neutrons), nuclear magnetic resonance (NMR) spectroscopy and electron cryo-microscopy (3DEM) methods. The Worldwide PDB (2) (wwPDB, wwpdb.org) was formed in 2003 to ensure global management of the PDB archive for the public good. The wwPDB founding members were three wwPDB regional data centers: the Research Collaboratory for Structural Bioinformatics PDB (RCSB PDB) (1) in the United States, the PDB in Europe (PDBe) (3) and PDB Japan (PDBj) (4). The Biological Magnetic Resonance Bank (BMRB, University of Wisconsin in USA and Osaka University in Japan) (5), which manages an archive of NMR experimental data, joined the wwPDB in 2006.

PDB data from MX, NMR and 3DEM are accessed by both non-expert and expert data users globally. In 2016, >1 million data users worldwide performed >590 million structure data file downloads, corresponding to ∼1.5 million data downloads per day. Based on our analysis of the annual Database issues of *Nucleic Acid Research* from 2011 to 2016 (www.oxfordjournals.org/nar/database/a/), ∼200 data resources access and use PDB data. The PDB archive is accessed by a large and diverse user community that encompasses researchers working in biotechnology, agricultural and pharmaceutical industries, academic and public sector scientists, students, educators, the curious public, with >80% of users having no or limited expertise in structural biology. PDB data and resources are used for basic and applied research across the sciences and in education, textbook publishing, experimental and computational methods development, drug discovery to name but a few.

Biocuration is central to PDB data management. Indeed, the PDB archive is widely regarded as one of the best-curated biological data resources available (6). The primary goal of biocuration is to accurately and comprehensively represent biological knowledge, to enable computational analysis and to provide easy access to data for scientists, educators and students. It involves translation, standardization and integration of information relevant to biology into a data archive or resource, thereby enabling integration with the scientific literature and management of large data sets (www.biocuration.org/dissemination/who-are-we/). All data submitted to the PDB undergo critical review by subject matter experts who curate, annotate and validate incoming data for completeness and accuracy.

Currently, in addition to 3D atomic coordinates, each PDB data deposition contains experimental data and metadata describing the molecular model and experimental details. Metadata encompasses protein names, sequences, source organism(s), small-molecule information (e.g. chemical name, structure and formula), data collection information (e.g. instrumentation and data processing) and structure-determination information (e.g. model-building, refinement and validation methods and statistics). In addition, the wwPDB provides value-added annotation such as secondary structure, quaternary structure descriptions and information about ligand-binding sites.

OneDep (7) is a unified tool that supports deposition, validation and biocuration and is used by both wwPDB Biocurators (hereafter *Biocurators*) and PDB data depositors (hereafter *Data Depositors*). It was developed by the wwPDB partners in collaboration with EMDataBank partners (8) to ensure that high-quality, internally consistent data are collected and that both the *Data Depositor* experience and biocuration processes are consistent worldwide. Introduction of OneDep has eliminated many sources of inconsistency that inevitably arose while wwPDB regional data centers were using independent data-processing systems. Occasionally, these differed in requirements for mandatory data items, different software and validation standards and distinct output data formats. To ensure consistency of data representation, OneDep uses the PDBx/mmCIF (9) data dictionary, which enables data standardization, data-model extension and seamless data exchange among wwPDB regional data centers. During development of OneDep, the wwPDB partners agreed on common practices for PDB data deposition, biocuration and validation. The OneDep system and wwPDB validation processes have been described in recent publications [Young *et al.* (7) and Gore *et al.* (10), respectively]. In this publication, we describe in detail the processes, practices and tools that wwPDB regional data centers employ during biocuration of PDB structure deposition.

## Importance of biocuration

### Data representation

The PDB has aimed to adhere to the *FAIR* principles (*Findability, Accessibility, Interoperability* and *Reusability*) (11) since its inception in 1971. Together, the wwPDB partners manage the archive and provide PDB data users (hereafter *Data Consumers*) around the world with unrestricted access to the structural data stored therein without limitations on data use (i.e. Reusability). To ensure Findability, each PDB entry is assigned a globally unique and persistent identifier. To ensure Interoperability with other data resources, PDB structure data, experimental data and associated metadata now conform to controlled vocabularies and semantic relationships defined in the PDBx/mmCIF dictionary (mmcif.wwpdb.org), which continues to be developed in collaboration with the scientific community (www.wwpdb.org/task/mmcif). In 2011, the PDBx/mmCIF format superseded the legacy PDB format flat file (9), originally used to store and distribute data. As of October 2017, the PDBx/mmCIF data dictionary encompassed almost 7000 data items, pertaining to atomic coordinates, experimental data, sample characteristics, structure-determination protocols, etc. The increasing size and complexity of macromolecular structures determined by MX, NMR and 3DEM, and introduction of new experimental methods have necessitated myriad changes to the PDBx/mmCIF data dictionary since its introduction. The data dictionary has also been augmented in response to the evolution of the PDB archive. The wwPDB PDBx/mmCIF working group (www.wwpdb.org/task/mmcif) works with wwPDB partners to ensure that this process is an orderly one. Biocurators work closely with Data Depositors to maintain data consistency and conformity with the PDBx/mmCIF data dictionary throughout the deposition process. As the PDBx/mmCIF dictionary evolves, *Biocurators* undertake periodic remediation of the contents of the PDB archive. Working as a global team, the *Biocurators* help to ensure that PDB data are indeed *Findable, Accessible, Interoperable* and *Reusable* for all *Data Consumers* worldwide.

### Data standardization

**Figure 1.**
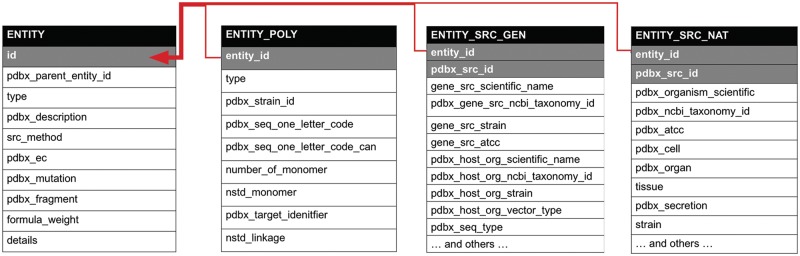
Semantic relationships in the PDBx/mmCIF dictionary. The partial diagram shows the relationships within an entity, its polymer sequence, source taxonomy and the method used to produce it. The relationships in these categories are described by a shared key identifier in a parent/child relationship as denoted with gray shading. The dictionary is available at mmcif.wwpdb.org/.

Data standardization is central to successful data resource management, as it ensures consistency and all of the benefits flowing therefrom (e.g. *Interoperability*). During standardization, data are brought to semantic integrity and to a common format that facilitates usability (and *Reusability*) and permits large-scale data analyses and distribution. Lack of standardization can result in incomplete, inconsistent and erroneous data retrieval, and thereby impede interpretation. Critical aspects of data standards, such as semantic consistency, use of controlled vocabularies and data-format consistency, are described in Figure 1 and Table 1. One of the most important components of the wwPDB biocuration process is ensuring that stored data are defined precisely and uniformly in machine-readable format, as defined by the PDBx/mmCIF data dictionary. The dictionary has a self-defining format in which every data item has attributes describing its features, including relationships to other data items, and supports validation of data items by providing controlled vocabularies and data types and ranges.

**Table 1.** Example of how the PDBx/mmCIF dictionary is used in describing the expression of a protein from *M. musculus* in *E. coli*

Dictionary item	Definition	Value
_entity_src_gen.entity_id	Unique identifier for each entity	1
_entity_src_gen.pdbx_gene_src_scientific_name	Scientific name for source organism	*M. musculus*
_entity_src_gen.gene_src_common_name	Common name for source organism	Mouse
_entity_src_gen.pdbx_gene_src_ncbi_taxonomy_id	Taxonomy id for source organism	10090
_entity_src_gen.gene_src_strain	Strain of the source organism	
_entity_src_gen.pdbx_host_org_scientific_name	Scientific name for expression host	*E. coli*
_entity_src_gen.pdbx_host_org_ncbi_taxonomy_id	Taxonomy id for expression host	562
_entity_src_gen.pdbx_host_org_vector_type	Type of vector used for expression	plasmid
_entity_src_gen.plasmid_name	Name of plasmid used for expression	pET28a

Note the use of the _entity_id key that links this category to the entity category as depicted in Figure 1. id, identifier.

Controlled vocabularies are used throughout the PDBx/mmCIF data dictionary to minimize ambiguity. About 600 mmCIF items in the PDB archive have enumeration lists, which are enforced during the deposition and biocuration processes (e.g. polymer types, entity types, instruments used in data collection and names of software packages). Such enumerations are extended as needed to keep pace with scientific and technological innovation. Although the dictionary can establish syntax for values, manual biocuration is often required to ensure accuracy and adherence to wwPDB policies. For example, polymer sequence, organism taxonomy, quaternary structure and ligand chemistry require expert manual inspection to validate correctness, scientific accuracy and internal consistency.

Semantic consistency represents another critical aspect of data standardization, helping to eliminate ambiguities in understanding existing data items. For example, Figure 1 shows the category relationships for a molecular entity that ensure semantic consistency with the PDBx/mmCIF data dictionary. The ‘entity’ category represents a unique polymer or non-polymeric constituent in the entry and is the key for ‘_entity_poly’, which describes the sequence of the polymer, and for ‘_entity_src_gen’ or ‘_entity_src_nat’, which show how the polymeric entity was produced: genetically manipulated or naturally occurring, respectively. The relationships in these categories are described by a shared key identifier in a parent/child relationship (denoted with gray shading in Figure 1). Table 1 provides an example of ‘_entity_src_gen’, which describes a protein from *Mus musculus* produced by heterologous expression of the mouse gene in *Escherichia coli*.

### Data quality control

To enforce data standardization, consistent relationships with multiple biological resources are maintained. Multiple external data resources are used and cross-referenced by the OneDep system, including the National Center for Biotechnology Information (NCBI) (12) taxonomy database and the UniProt (13) sequence database. For example, the controlled vocabulary for organism name employed within the OneDep deposition user interface corresponds to that of the NCBI taxonomy database, while protein sequences are mapped to the appropriate UniProt identifier on the basis of the taxonomy and other information supplied by the *Data Depositor*.

The OneDep system also controls data quality by setting boundaries for data values. These limits are determined according to scientific principles or by examining distributions of existing data items in the archive. Some data items have ‘hard’ limits (e.g. pH value or absolute temperature), while others have ‘soft’ limits (or likely ranges), such as *R*-values in MX. These limits are maintained in the PDBx/mmCIF data dictionary, and outliers are reported during OneDep deposition, validation and biocuration. Soft limits are provided for many items that follow a normal distribution, with values more than three standard deviations from the mean are noted as outliers to *Data Depositors* who are asked to check and correct the value if necessary. For example, the boundary for the observed *R* value for merging intensity (Rmerge) is set between 0.01 and 0.2 for soft limits. The system will therefore flag this data item as a warning if the provided value is 0.7 or 13 when a value of 0.13 is expected.

### Role of expert manual biocuration

PDB data are curated by professional *Biocurators*. The 17 *Biocurators* currently working across the wwPDB have, among them, strong domain expertise in MX, NMR, 3DEM, chemistry, biochemistry and molecular biology. Their primary responsibilities are to examine/validate incoming data (in collaboration with *Data Depositors*) to maintain the quality of the PDB archive and to release these data in a timely manner. They also regularly review PDB archive contents and perform remediation to improve data uniformity, quality and consistency.

*Biocurators* check deposited data for completeness, self-consistency and accuracy. For each incoming structure, they assess information about all steps in the structure-determination process, from protein expression, crystallization, sample preparation and data collection to final model refinement, resolving conflicting information and providing *Data Depositors* and *Data Consumers* with a comprehensive description of the structure. Despite automation of many processes, there are significant points where 3D structure data biocuration requires manual inspection, extensive scientific knowledge (particularly for ligand and sequence processing) and sometimes requires dialog with the *Data Depositor*.

With the OneDep system, *Biocurators’* workloads are balanced using automated geographic distribution on the basis of *Data Depositor* location. Of the 11 641 global depositions received in 2016, RCSB PDB processed ∼45% (coming mainly from the Americas and Oceania), PDBe processed 36% (Europe and Africa) and PDBj processed the remaining 19% (Asia). Geographic distribution has enabled *Biocurators* to communicate more efficiently with *Data Depositors*, with the majority of *Data Depositors* located in similar time zones as the wwPDB regional data center handling their submissions.

It is critical that *Biocurators* communicate among themselves to develop and standardize common biocuration practices and policies, to resolve annotation issues and to set functional requirements for improvements in the OneDep system to ensure high data quality, thereby contributing to the success of the wwPDB. Beyond day-to-day local interactions, this international team communicates through daily emails, weekly virtual meetings and annual face-to-face meetings. wwPDB biocuration policies and procedures are fully documented (wwpdb.org/documentation/annotation).

The wwPDB has long-standing relationships with many journals, allowing coordination of PDB data release in the public domain with the appearance of the corresponding scientific publications. wwPDB policies stipulate that PDB structure data should be publicly available when the structure-determination report is published, either electronically or in print. A number of journals inform the wwPDB on a weekly basis about upcoming articles and provide corresponding PDB IDs, publication dates and citation information to ensure nearly simultaneous publication of the research and release of corresponding PDB structure data. Current wwPDB policies stipulate that depositions should not be withheld from public release for more than 1 year from the time of submission and that depositions are to be released upon publication of a relevant article. If no publication appears within the 1-year period, the deposited structure must either be released or withdrawn.

Many journals now require that authors submit the official wwPDB validation report as part of the article submission/review process. These reports provide information about structure quality and various analyses of experimental data (10). They are frequently used by referees to confirm the accuracy and quality of the work under review. Currently, wwPDB validation reports are required for article submission by the Nature Publishing Group journals, eLife, the Journal of Biological Chemistry, International Union of Crystallography (IUCr) journals, Structure, Federation of European Biochemical Societies journals, the Journal of Immunology and Angewandte Chemie International Edition. Others strongly encourage submission of wwPDB validation reports with articles.

Every *Biocurator* also participates in outreach, education and public engagement activities to serve structural biologists, other researchers, educators, students, schools and the curious public. The wwPDB maintains a customer service desk for *Data Depositors* and *Data Consumers*, receiving communications from around the world. Sometimes, these communications report errors and/or *Depositors*’ corrections helping to improve the quality of the archive. PDB users often notify the wwPDB when a structure-determination report has been published to help trigger public release of relevant data into the PDB archive.

## wwPDB biocuration methodology

### Biocuration by Data Depositors

Biocuration begins at the time of structure deposition through the OneDep system. Mandatory data items are validated against the PDBx/mmCIF data dictionary for format compliance and completeness. A valid PDB deposition provides not only primary data and associated metadata but also critical information that helps *Biocurators* properly annotate the structure without relying solely on the atomic coordinates. For example, every deposition must include information about polymer sequences, quaternary structure and ligands present in the PDB entry.

Many structure-determination studies focus on one or more bound ligands (chemical components), including drugs, inhibitors or substrates. The *Data Depositor* has the option of identifying each such ligand as a ‘ligand of interest’. In cases where the connectivity, bond orders and chirality of the ligand do not exactly match an existing entry in the wwPDB chemical component dictionary (CCD) (14), *Data Depositors* are asked to provide additional chemical information to ensure accurate identification of each ligand. This information must include at least one of either a chemical drawing, a SMILES string, an appropriate CCD reference identifier or the ligand restraint file that was used during structure refinement. This information is particularly important when the ligand was not built in its entirety during structure determination, where a tautomeric ligand is present, or where correct geometry and bond order cannot be inferred readily from the atomic coordinates.

*Data Depositors* are required to provide the sequences of all unique amino acid and nucleic acid macromolecules present in the experimental sample, and they are required to reconcile these sequences with the sequences represented within the atomic coordinates. *Data Depositors* are encouraged to provide sequence database references (e.g. UniProt), together with a description of any deletions, insertions, engineered mutations or affinity purification tags present in experimental samples.

For higher order quaternary structures or assemblies (e.g. dimers, trimers and tetramers), *Data Depositors* are expected to identify assemblies present in the experimental sample and provide any geometric transformations necessary to generate the corresponding quaternary structure from the crystallographic asymmetric unit (e.g. apply a symmetry matrix to the coordinates of a protein chain to generate a dimer). *Data Depositors* are also able to provide information regarding any experiments used to determine the quaternary structure in solution as supporting evidence to be included in the PDBx/mmCIF archival data file.

Once primary data have been uploaded and harvested, the OneDep system generates a preliminary wwPDB validation report, which identifies potential issues with the structure and/or experimental data. Before concluding the submission process, *Data Depositors* are required to download and review this validation report, and either to accept the report as is or choose to improve the deposition by uploading revised data. *Data Depositors* are strongly encouraged to correct any issues prior to finalizing the deposition. Once the validation report and the terms of wwPDB submission are accepted, the *Data Depositor* can submit the data. At this point, PDB, BMRB and/or Electron Microscopy Data Bank accession codes are issued and the deposition is transferred for internal processing by *Biocurators*.

### Biocuration by wwPDB Biocurators

**Figure 2.**
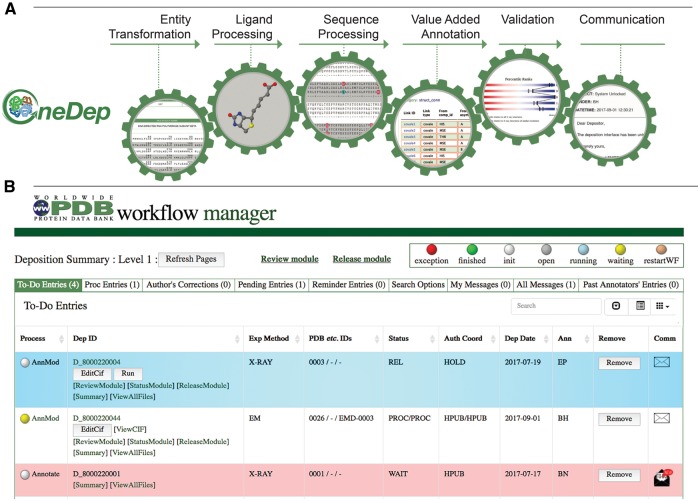
Major modules controlled by the workflow and WFM. (**A**) Mandatory major tasks controlled by the workflow with a pre-defined order. (**B**) WFM interface that allows Biocurators to prioritize their tasks, to manage multiple entries and to access module user interface for manual biocuration after completion of automated calculations.

The wwPDB biocuration workflow has been designed to execute *mandatory* tasks automatically and invoke other necessary tasks on demand. Based on extensive combined biocuration experience across the wwPDB, a series of mandatory and optional tasks have been identified and organized into several modules within the OneDep workflow as shown in Figure 2A. Each module is initiated upon successful completion of the previous module in the workflow. Because proper execution of many tasks is dependent upon successful completion of previous tasks, the workflow system ensures that all tasks are performed in the correct order. Some tasks require extensive review and/or input from *Biocurators*; others can be performed automatically.

The wwPDB biocuration workflow is controlled *via* an interactive workflow manager (WFM), which informs *Biocurators* when an automatic process has finished. For example, when an automated Proteins, Interfaces, Structures and Assemblies (PISA) calculation for quaternary structure prediction is completed, the workflow status changes from gray to yellow color informing Biocurators that they can access user interface in the value-added annotation module for further manual biocuration, as shown in Figure 2B. The system also allows *Biocurators* to monitor progress of multiple entries, access each module for inspection and perform manual curation of entries. The WFM tracks and logs completion of modules and provides *Biocurators* with the ability to restart processing at any point along the workflow or run individual modules outside of the normal workflow. The WFM manages correspondence with *Data Depositors* and signals whether sent messages have been read or require a reply. If a PDB deposition needs to be updated by the *Data Depositor*, the *Biocurator* can unlock the deposition interface, suspending further biocuration until appropriate *Depositor* action has occurred. Entries ready for release are highlighted by the WFM.

Following initial content review, *Biocurators* begin with the entity transformer module, which surveys the overall polymer *versus* non-polymer (ligand) representation. This is followed by instantiation of the ligand processing module to check ligand stereochemistry and assign the correct ligand reference identifier (CCD three-letter code). Thereafter, the sequence processing module enables cross-referencing of polymer sequences and taxonomy. Finally, the *Biocurator* provides value-added annotation with the aid of the annotation module. Once annotation of a PDB entry is complete, *Biocurators* use the validation module to assess the quality of the atomic structure and its agreement with experimental data. At the end of the biocuration process, *Biocurators* use the communication module to compose a letter (highlighting major issues), which is then sent together with the processed files and a validation report to the *Data Depositor* for approval or correction. The automated workflow tasks and manual biocuration tasks for each module are described in Table 2. Modular biocuration steps are described in further detail later.

**Table 2.** High level description for each module of automated tasks performed by workflow and manual tasks performed by wwPDB *Biocurators*

Workflow modules	Automated calculations	Manual biocuration tasks
Entity transformation	Sequence similarity search Structure similarity search Split or combine polymer entities PRD identification	Review search hits Decide whether polymer entities should be split or combined PRD assignment
Ligand processing	Batch sub-graph search of CCD Calculate matching score Generate 2D and 3D images	Review the closest match or no match instances Visually inspect covalent linkage with proteins Assign ligand to the closest matched CCD Create new ligand definition
Sequence processing	Sequence search (BLAST) against UniProt or GenBank Calculate the score and rank the hits Perform and display multiple sequence alignments List alignment discrepancies from the reference	Select proper sequence reference Visually review sequence alignments Annotate sequence discrepancies Annotate sequence fragments for chimeric proteins
Value-added annotation	Run PISA for quaternary structure prediction Solvent repositioning for X-ray entries Nomenclature mapping in chemical shifts for NMR entries Map parameters annotation for 3DEM entries Generate interatomic connectivity (links) Generate secondary structures Run various checks	Visualize 3D quaternary structure and select most likely assemblies Annotate meta data Review connectivity and add or delete as appropriate Review electron-density fit to the model for X-ray ligands
Validation	Run wwPDB validation software Generate validation XML and PDF reports Generate correspondence letter template	Review validation PDF report Highlight major issues in the correspondence letter
Communication	Provide letter template Copy annotated PDB files Send validation report, PDB files and correspondence letter to deposition interface Notify depositors to review files Reset deposition interface to reflect annotated data	Attach additional materials as needed Activate sending files to deposition interface

BLAST: Basic Local Alignment Search Tool; PDF: Portable Document Format.

#### Initial review

Upon initiation of the OneDep workflow, the report module analyses the data for errors and/or inconsistencies. The report module generates an internal report that includes both the results of these calculations and a listing of selected metadata. This initial review informs the *Biocurator* about the content of the deposition and highlights issues that may need to be examined and, if possible, corrected during processing.

#### Entity transformation

Within the PDB entry there may be multiple instances of a particular chemically distinct molecule, referred to as an entity (first module in Figure 2A). As discussed earlier, entities may be polymers (e.g. protein or nucleic acid) or non-polymers (e.g. organic ligands, ions or solvent molecules). Ligands covalently bound to polymers are usually defined as non-polymer entities independent of the polymers to which they are attached (with the exception of some common post-translationally modified residues).

The entity transformation module enables *Biocurators* to ensure that the ligands in a newly deposited structure are depicted in a manner that is consistent with others already present in the PDB archive. In some cases, the ligand as provided by the *Data Depositor* may need to be described in terms of smaller components. For example, a peptide-like small molecule, such as some antibiotic compounds, may be treated as a string of modified and/or unmodified amino acids, if the constituent parts adhere to the rules that designate a polymeric entity, or as a large ligand (non-polymer). Whereas a non-polymeric representation is usually convenient for defining overall connectivity and restraints during structure determination and refinement, polymeric representations are typically better at depicting the underlying biochemistry. Although each type of representation has intrinsic benefits, it is important to ensure their consistent representation in the atomic coordinate files across the PDB archive. Peptide-like small molecules were exhaustively reviewed in 2012, and since then have been represented consistently in both the CCD and atomic coordinate files. This process included introduction of an additional representation to describe peptide-like ligands, called the peptide reference dictionary (PRD), to retain an overall definition for peptide-like small molecules (15).

The entity transformation module searches the atomic coordinate file of the newly deposited structure and returns close peptide-like small molecule matches in the CCD and PRD. The interface allows *Biocurators* to compare polymeric sequences and 2D and 3D atomic configurations of ligands with matched PRD definitions. This module also includes tools that allow transformation between non-polymer and polymer representations to ensure consistency across the PDB archive. In addition to changing how ligands are represented, polymer chains may need to be split or merged depending on whether or not they are covalently linked *via* a standard peptide bond or nucleic acid linkage. Since re-configuration of polymers and non-polymers often requires repeating the biocuration process of either ligands or polymer sequences (or both), it is important that all entity types are properly defined at the outset.

#### Ligand processing

Structures of ligands bound to biological macromolecules provide atomic level insights to aid understanding of the function of protein molecules, aid in drug discovery and serve other research applications. About 75% of all structures currently in the PDB archive contain at least one ligand that is not a water molecule. Hence, ligand processing (16, 17), involving verification of chemical identity, validation of geometrical quality and validation of atomic coordinates against experimental data, is one of the most important aspects of wwPDB biocuration. Verification of chemical identity involves matching of all instances of a given ligand within a newly deposited structure to a corresponding chemical definition in the CCD (14), and standardization of atom naming to conform to the nomenclature defined in the CCD.

**Figure 3.**
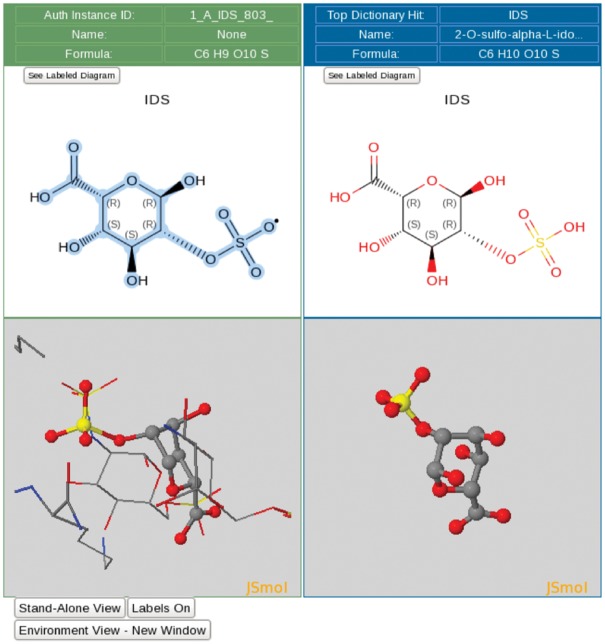
Ligand processing. The ligand processing module enables comparison of the structure of the deposited ligand with matches from the CCD. The top panels compare 2D structures, and the bottom panels compare 3D views of the model with matched ligands.

The ligand processing module extracts all non-polymer entities and non-standard polymeric residues from the deposited atomic coordinates and performs a sub-graph isomorphism search of the CCD. This search returns a list of top hits ranked by the matching scores and provides interactive 2D and 3D ligand views that allow visual inspection of both the *Data Depositor*-provided ligand structure and the corresponding matched CCD component (Figure 3). Additional chemical information (e.g. SMILES string), if provided by the *Data Depositor*, is illustrated in a 2D chemical drawing for *Biocurator* verification. If no match to an existing CCD entry is found, the *Biocurator* defines a new chemical component for the CCD using the ligand editor functionality of the ligand processing module.

Standardization of atom nomenclature to that in the CCD is a fully automated process but match identification is considerably more complex and often requires *Biocurator* review and manual intervention. The *Biocurator* notifies the *Data Depositor* of any problems regarding ligand identity, configuration and conformation. Typical steps followed during ligand biocuration may include but are not limited to:

- Reconciliation of additional ligand information provided by the *Data Depositor*, [e.g. CCD IDs, SMILES strings, International Union of Pure and Applied Chemistry (IUPAC) names or images, with ligand instances] present in the entry.
- Recognition and identification of existing CCD components, even in cases where portions of a ligand are not modeled, or where geometric errors (e.g. incorrect chirality, bond lengths and angles or bond orders) are detected. The *Biocurator* is able to review the chemistry within this module but may also need to review literature sources, consult with online resources (e.g. PubChem) or communicate with *Data Depositors*.
- Creation of new ligand definitions when a match is not present in the CCD. Since a new CCD component will be used as a reference for all future PDB depositions containing the same ligand, *Biocurators* invest a considerable effort to verify the chemical identity of each ligand. In many instances, *Biocurators* seek confirmation from *Data Depositors* or confer with other members of the global wwPDB biocuration team.
- Notification to *Data Depositors* of problems regarding ligand identity, configuration and conformation.
- Verification of interactions with the macromolecular host.

Redundancy and consistency checks among PRD, CCD and PDB entries are performed, and the *Biocurator* is alerted to any discrepancies found between the newly deposited atomic model and any of these resources. Examples of such discrepancies include a peptide-like small molecule present in the deposition that is not referenced to an existing PRD entry or a non-polymer ligand in the deposition that should have been represented as a polymeric peptide according to the PRD.

#### Sequence processing

This module compares the amino acid or nucleic acid polymer sequence provided by the *Data Depositor* to both the sequence represented within the deposited atomic coordinates and a sequence from an external reference database such as GenBank (12) or UniProt (13). wwPDB policy requires that *Depositors* report the actual polymer sequences of the molecules present in the experimental sample, including any modifications or added portions (e.g. engineered mutations, post-translational modification, affinity tags for purification and cloning artifacts). In addition, the deposited information must include any segments of the polymer chain that were not included (for any reason) in the atomic coordinates but which were present in the experimental sample (e.g. unmodeled loop regions). Moreover, there should be no discrepancies between the deposited sequence(s) and the atomic coordinates. The source organism for the deposited sequence (naturally obtained or engineered) should be provided, with the exception of non-biological sequences which have the source organism identified as ‘synthetic construct’. If the deposited polymer sequence is consistent with a reference sequence entry from UniProt (for proteins) or GenBank (for nucleic acids), then the corresponding accession from these databases is captured and any discrepancies between the sample sequence and the reference are annotated. These mandatory elements are necessary but not sufficient to complete sequence annotation.

Sequence comparison [BLAST (18)] is run automatically against UniProt (for proteins) and GenBank (nucleic acids), with the result used by the *Biocurator* in conjunction with sequence identity and taxonomy matching to determine the appropriate cross-reference. In some cases, further clarification is required from the *Data Depositor* as to the exact content of their experimental sample. Comparisons between the experimental sequence, the sequence derived from the atomic coordinates and sequence database results are used to identify affinity tags (or cloning artifacts, depending on the *Data Depositors’* description), insertions, linkers, deletions, possible mutations or variants and start and end points of segments within chimeric constructs. Visual inspection of alignments of the deposited sequences and the reference sequences from UniProt or GenBank allows identification of any peptide and/or nucleic acid linkage issues within the atomic coordinates and identification of incomplete experimental sample sequences provided by the *Data Depositor*. Sometimes mismatches between the sequences reflect errors in the deposited atomic coordinates. Such cases require that *Biocurators* consult with *Data Depositors* for clarification and/or correction. The reference sequence also helps *Biocurators* identify and annotate chimeric constructs (i.e. those derived from multiple source organisms). After sequence alignment verification, residues that are missing some of their sidechain atoms and any residues labeled incorrectly as alanine or glycine are updated to match the sample sequence. External sequence references are also used to standardize the protein name, the scientific name of the source organism and its taxonomy.

**Figure 4.**
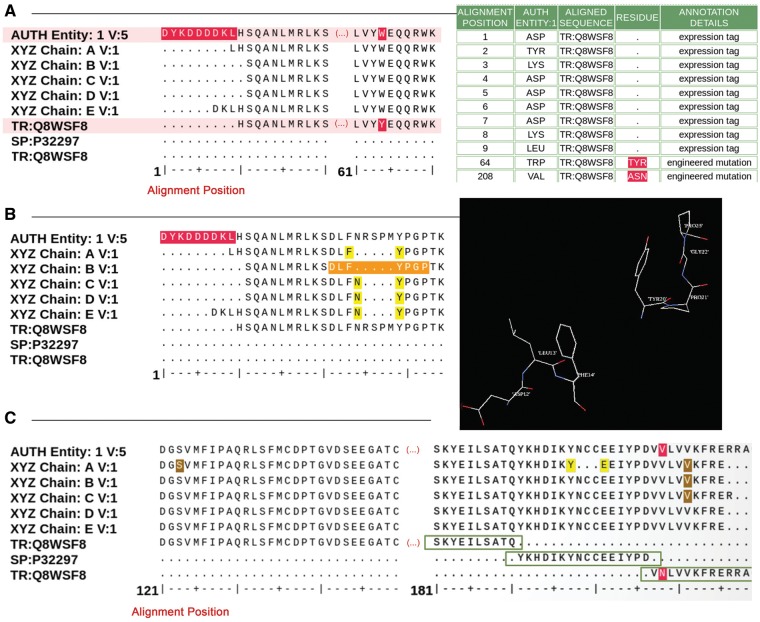
Examples of sequence alignments from the sequence processing module. (**A**) Alignment between the sample sequence (labeled ‘AUTH Entity’), the sequence extracted from the atom data for each polymer chain and the corresponding UniProt sequence. Sequence discrepancies are highlighted in red and listed in a table where the appropriate annotation can be selected. For example, residues 1–9 in the sample sequence (top sequence in the alignment) are not present in the UniProt sequence (bottom sequence in the alignment) because these are part of an expression tag. Similarly, the Tyrosine-Tryptophan (TYR-TRP) conflict at position 64 is annotated as a mutation based on information provided by the *Depositor* during data submission. (**B**) Example illustrating the sequence and 3D viewer. Residues depicted in orange in the sequence are highlighted and selected for visualization with the 3D viewer available on the alignment page. (**C**) Example of a sequence alignment for a chimeric protein construct. This chimeric acetylcholine-binding protein from *Aplysia californica*, PDB entry 5TVC, contains a loop C from the human alpha-3 nicotinic acetylcholine receptor. The alignment shows that residues 1–181 in the deposited sample sequence correspond to UniProt sequence Q8WSF8, residues 182–197 to UniProt sequence P32297 and residues 198–219 again to UniProt sequence Q8WSF8.

Figure 4 shows examples of sequence alignments used by *Biocurators* during sequence annotation. The *Data Depositor*-provided sample sequence, the sequences extracted from the atomic coordinates for each polymeric chain in the structure and closely matching UniProt sequences are aligned and presented for analysis. Discrepancies between the sequences are highlighted and listed in an interactive table, where *Biocurators* can select the appropriate annotation from a controlled vocabulary list (Figure 4A). If any part of the structure requires visual inspection, *Biocurators* select the relevant residue range and use the 3D viewer available within the sequence processing module to examine the 3D structure of the corresponding sequence range (Figure 4B). This feature is particularly helpful for inspecting sequence connectivity and alignment, particularly for disordered or poorly resolved regions of a structure where residues or sidechains were omitted from the deposited atomic coordinates. Figure 4C illustrates the case of a chimeric protein containing a fusion of partial sequences from two different proteins that align with sequences from distinct UniProt entries. Correct sequence annotation for chimeric proteins requires inclusion of the residue range and source organism name for each segment of such a chimera. If this information is not provided during deposition, *Biocurators* will request it from the *Data Depositor*.

#### Value-added annotation

**Figure 5.**
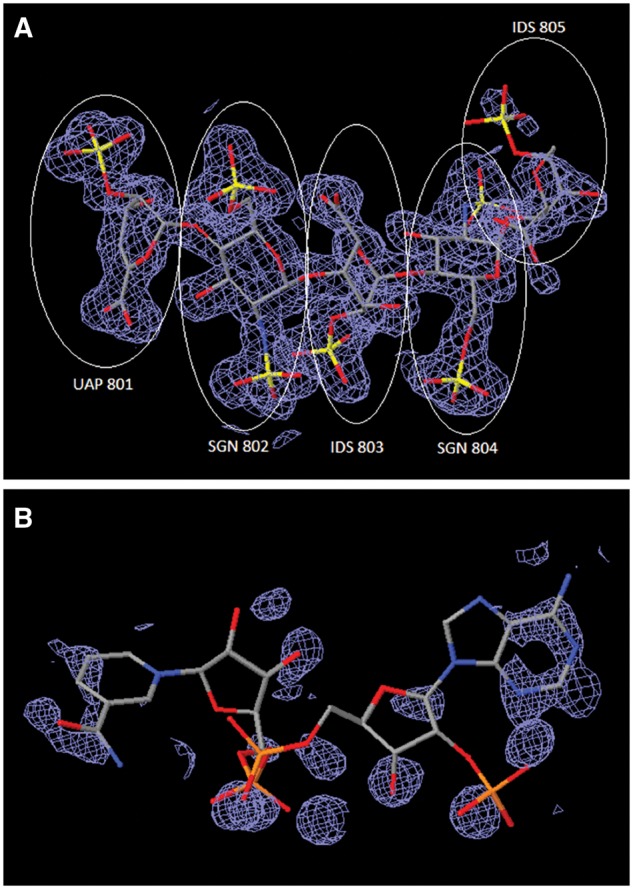
Comparison of ligand structures with 3D electron-density views. The electron-density maps shown in Figure 4A and B are 2m|Fo|-D|Fc| maps contoured at 1.0 σ cutoff. (**A**) Good electron-density fit for heparin oligosaccharide at residues 801–804 bound to annexin in PDB entry 2HYV. (**B**) Poor electron-density fit for NADP bound to alcohol dehydrogenase in PDB entry 1ZK4.

The added annotation module of the OneDep system enables a series of automated calculations (tasks numbered i–v later) and semi-manual annotations of metadata (tasks numbered vi–viii):

- *Ligand and solvent chain associations and numbering:* Ligand and solvent chain identifiers and residue numbering are re-assigned automatically according to wwPDB policy where necessary.
- *Solvent position:* In MX structures, water molecules are moved to symmetry-related positions to place them closest to the polymer chains comprising the asymmetric unit. For water molecules that cannot be repositioned close to any polymer chain, *Biocurators* consult with *Data Depositor*s.
- *Links:* Interatomic links between any non-standard or polymeric residues and ligands are automatically generated and made available for *Biocurators* to review and correct as needed.
- *Secondary structure:* The OneDep system calculates protein secondary structure (19) for use by visualization programs that rely on PDB secondary structure records. On occasion, a *Data Depositor* may elect to provide secondary structure; in such cases, *Biocurators* incorporate this information and label the data as being author-determined.
- *Extended checks:* Although the official wwPDB validation report (10) is produced in a subsequent OneDep module, a series of tests are performed to evaluate the atomic coordinates and their fit to the experimental data. In addition to ensuring adherence of deposition contents against the PDBx/mmCIF data dictionary, results of additional scientific checks are provided for review (e.g. peptide-bond linkages, close contacts, unusual metadata values or inconsistent metadata values across different items). If *Biocurators* cannot correct identified issues, they consult the *Data Depositor*, and remaining issues may be highlighted in the final wwPDB validation report.
- *Quaternary structure (assembly) determination:* By convention, atomic structures determined by MX deposited into the PDB encompass only the smallest possible representation of the molecular component(s) comprising the crystal lattice (i.e. the asymmetric unit that repeats to form the crystal). These asymmetric units may constitute only a portion of the macromolecular assembly present in the experimental sample. In such cases, both the atomic structure of the asymmetric unit and applicable geometric transformations (rotation/translation operators) are required to generate computationally the atomic structure of the macromolecular assembly in its entirety. Uncertainties concerning the correct choice of macromolecular assembly from MX structures are not unusual. For example, there may be more than one energetically favorable spatial arrangement of asymmetric units, each corresponding to a distinct assembly. Without additional experimental evidence, it is generally not possible to determine which, if any, of these putative assemblies are relevant, or even occur in solution or *in vivo*. OneDep collects experimental evidence that supports assembly provided by the *Data Depositor*. Determination of possible macromolecular assemblies from the results of an MX structure determination is a complex multi-step process. First, assembly information provided by the *Data Depositor* is considered. Second, PISA (20) software is used to predict assemblies, which are cross-checked against the information provided by the *Data Depositor*. For viruses and other complex assemblies with point or helical symmetry, depositor-uploaded symmetry matrices are processed using the Pointsuite tool (21).
- *Metadata editor:* Common and experimental method-specific views are provided for metadata annotation using PDBx/mmCIF data dictionary controlled vocabularies.
- *Method-specific features:* Method-specific tools enable adjustment of metadata in both atomic coordinate and experimental data files. For MX depositions, e.g. the reported X-ray or neutron wavelength in the structure factor file is often misreported and can be corrected. For NMR, tools enable manipulation of chemical shift data files to ensure that their atom nomenclature is consistent with that of the atomic coordinates. For 3DEM, *Biocurators* can edit 3DEM map headers after checking the 3DEM maps themselves to ensure internal consistency with the other uploaded files; *Biocurators* also check the fit of the atomic coordinates to the 3DEM maps. Currently, this step is performed visually using The University of California, San Francisco Chimera (22) graphics display software. In addition, 3D interactive difference electron-density maps of ligands for MX entries are provided at different contour levels for *Biocurators* to verify structural details. For example, Figure 5A displays the electron-density fit of heparin oligosaccharide bound to annexin in PDB entry 2HYV (23). This case is an example of a good electron-density fit for four well resolved monosaccharides (residues 801–804) and with partial density fit for a disordered monosaccharide (residue 805). Figure 5B illustrates an example of poor electron-density fit for the Nicotinamide adenine dinucleotide phosphate (NADP) ligand bound to alcohol dehydrogenase in PDB entry 1ZK4 (24). The wwPDB validation report ligand-related statistics for this entry include an extremely high real space *R*-factor value (25, 26) of 0.67 for the NADP ligand. Detailed analyses of this particular case were reported by Weichenberger *et al.* (27) and Shao *et al.* (28).

#### Validation/final review

An important goal of data quality control through biocuration is to ensure that the interpretation of the experimental data is consistent. At the end of the OneDep biocuration pipeline, a wwPDB validation report (10) is generated for the *Data Depositor*. This document, which was developed in collaboration with community experts (29–31), serves as the official wwPDB validation report that the *Data Depositor* is strongly encouraged to provide to scientific journals to aid article review.

The wwPDB validation report highlights any unusual geometric features within the atomic coordinates. For MX structures, the report also highlights any discrepancies between the atomic coordinates and the experimental data from which the structure was determined. The report is separated into sections that describe polymer and non-polymer components. Outliers are highlighted in tabular form within the report and are also shown in the form of a high-level summary. The validation measures of the deposited structure are compared with those of similar entries in the PDB and given a percentile score so that the *Data Depositor* (as well as journal editors and referees and subsequently *Data Consumers*) can see at a glance how the quality of this structure compares to that of others in the archive.

#### Communication with *Data Depositors*

The OneDep communication module enables all communication between *Data Depositors* and *Biocurators*, for a particular deposition, to be archived in one place. Once the wwPDB biocuration process is complete, *Biocurators* summarize any outstanding issues in a standardized letter, much of which is generated automatically. This summary letter along with the atomic coordinates, experimental data and wwPDB validation report are all made available to the *Data Depositor* through the OneDep deposition user interface. The *Data Depositor* receives an email notification to log back into the OneDep system and review the curated data files and the wwPDB validation report. At this stage, corrections may be requested to remedy any major issues identified during biocuration, such as polymer chain breaks, stereochemical (chirality) errors in residues or ligands and interatomic clashes. Frequently, *Biocurators* also seek *Data Depositor* clarification on the sample sequence used in the experiment, annotation of the quaternary structure macromolecular assembly, ligands and inconsistent data items, etc. Timely response helps expedite completion of the deposition process and preparations for public release. On receipt of the *Data Depositor’*s response, *Biocurators* incorporate changes to the deposition and send updated files and validation reports back for review/approval. Once finalized, the new PDB entry is released in accord with the *Data Depositor’s* instructions and wwPDB policy.

*Data Depositors* are notified 3 months, 2 months and 1 month prior to the 1-year hold-expiration date. The PDB entry is released at the end of the 1-year period if the *Data Depositor* does not respond to the hold-expiration notification. The wwPDB is alerted to publication dates and citation information by *Data Depositors*, some scientific journals and frequently by *Data Consumers*. In addition, the OneDep citation tracker scans the literature for publications on a weekly basis. Once a citation has been found, the relevant *Data Depositor* is notified about the upcoming release date and the citation details.

## Outcomes

### Improved efficiency

**Figure 6.**
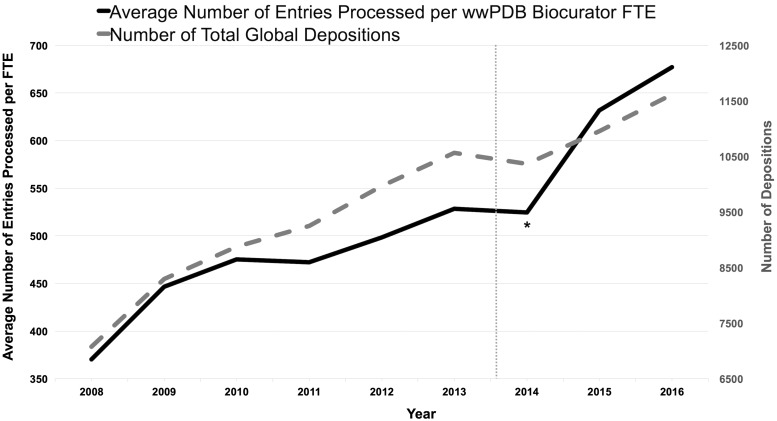
Average number of entries processed per wwPDB biocurator FTE and number of total global depositions per year. The processing productivity per wwPDB biocurator FTE has nearly doubled since 2008 as shown in this graph. This graph also reflects that the productivity was accelerated with the OneDep system. The label * indicates the transition period when both new OneDep and legacy systems were operated in parallel. The dash line indicates when the wwPDB validation report was first introduced in August 2013.

Figure 6 illustrates the average number of PDB depositions processed annually per *Biocurator* full-time equivalent (FTE) and the number of total global depositions as a function of time. This graph shows that productivity has nearly doubled since 2008, reflecting a regime of continuous improvement that was accelerated by the OneDep system. During the transition period (indicated as * in the Figure 6), productivity was not improved due to the OneDep system was first put into production, and *Biocurators* had to operate both new and legacy systems in parallel and were learning to use the new system. In addition, the total number of annual depositions fell slightly in 2014. Efficiency gains continued once the OneDep system was fully implemented and replaced the legacy systems. These productivity improvements come despite year-on-year increases in the complexity of the structure depositions (7) along with a significant increase in better-quality added-value such as ligand annotation, quaternary structure definitions and comprehensive validation report provided in the OneDep system. The dash line indicates the comprehensive wwPDB validation report was first introduced to *Depositors* prior to the OneDep system in August 2013.

Assisted by the OneDep system, *Biocurators* not infrequently identify issues with deposited data and request corrections from *Data Depositors*. Based on wwPDB correspondence records, the most frequently raised issues during biocuration are as follows: ligand chirality errors (∼26% of all issues raised), polymer backbone linkages (∼24%), interatomic clashes (∼12%) and sequence discrepancies between reference and *Data Depositor*-provided sequences (∼8%) as shown in Table 3. About 13% of the total number of depositions within a recent 6-month period had at least one of the issues listed in Table 3 raised by *Biocurators*.

**Table 3.** Common issues raised during biocuration ranked by frequency

Type of issue	Frequency of raised issue (%)
Chirality error	26
Polymer backbone linkage	24
Atomic clashes	12
Sequence discrepancy	8
Atoms with unrealistic or zero occupancies	8
High real space *R*-factor and low correlation in the structure factors	5
Incomplete data in the structure factors	3
Missing and/or inconsistent metadata values	3
Reported and calculated quaternary structure do not agree	2
Ligand geometry	1
Occupancy of atoms on special symmetry positions	1
Wavelength discrepancy	1
Missing anisotropic *B*-factor	1
Ligand identity	1
Distant waters	<0.5
Polymer geometry	<0.5
Unusual φ/ψ torsion angles	<0.5
Extra hydrogen atoms	<0.5
Zero *B*-factor	<0.5
Missing free *R* test set in the structure factors	<0.5

In the most serious cases, *Data Depositors* provide replacement data (atomic coordinates and/or experimental data). In 2015, 29% of depositions underwent data replacement (falling to 25% in 2016). Although time-consuming for *Biocurators*, the wwPDB regards this as a ‘good problem to have’. It also helps to inform on-going improvements to the OneDep system so that *Data Depositors* are alerted to potential issues as early as possible.

The wwPDB is committed to helping all *Data Depositors* improve data quality, while working to improve *Biocurator* efficiency. We, therefore, provide an anonymous wwPDB validation server for use prior to deposition and are working to make this facility as widely known as possible. We are collaborating with major structure determination and refinement software developers to promote use of the wwPDB validation webservice application programming interface so that *Data Depositors* can more easily validate their structures prior to deposition. We continue to improve the way in which the OneDep deposition module reports major issues to our *Data Depositors*, making it more likely that these issues will be addressed before the expert *Biocurators* begin their work.

### Improved data quality

Following introduction of the wwPDB OneDep system in 2014, data completeness has improved, as the number of data items in the dictionary that are mandatory has nearly doubled (2280 *versus* 1249 mandatory data items) since year 2014. In addition, depositions have become more consistent because of increased use of controlled vocabularies (596 *versus* 474 data items that have controlled vocabularies defined). Structures deposited using OneDep are also exhibiting higher data quality (10, 28). PDB *Data Depositors* and *Data Consumers* have become more aware of quality assessment since 2015, when wwPDB validation reports became available for the entire PDB archive. The OneDep system has also enabled better representation of chimeric proteins through complete annotation of each sequence fragment within a polymer entity.

The wwPDB is committed to maintaining uniformity and standardization across the entire archive. Data representation for newly determined structures can be challenging as methods in structural biology evolve and as the structures themselves become more complex. To address these challenges, *Biocurators* regularly review the archived data and perform archival updates (i.e. remediation) to improve data representation and ensure consistency. Data categories and items in the PDBx/mmCIF data dictionary are often extended or enhanced during remediation campaigns.

The wwPDB has undertaken several major archival remediation projects over the past decade. In 2007, efforts were made to standardize atom nomenclature, update sequence references and provide taxonomy information (32). In 2008, representation of icosahedral viruses was made uniform (21). In 2011, uniform/dual representation for peptide-like small molecules was accomplished (15). In 2014, very large structures, which were historically split into multiple PDB entries (due to the limitations of the legacy PDB file format) were combined into single files and entries in PDBx/mmCIF format. This measure allowed the remediated large structures to be visualized in 3D in their entirety and validated against experimental data for the first time. In 2017, the PDBx/mmCIF atomic coordinate files in the PDB archive were updated to conform to the latest version of PDBx/mmCIF data dictionary. In addition, the representation of chimeric proteins was standardized through complete annotation of each sequence fragment within a polymer entity. wwPDB remediation efforts are on-going to ensure consistency across the archive. Major wwPDB remediation undertakings have been reported in peer-reviewed scientific publications (15, 21, 32), and all wwPDB remediation activities are documented on the wwPDB website (www.wwpdb.org/documentation/remediation). Importantly, the OneDep system contains functionality to support remediation efforts, thus making them more efficient.

### Future challenges and conclusion

There are many challenges ahead that the wwPDB partners need to address.

1. Keeping pace with new developments in structure-determination techniques: New and evolving techniques in structural biology, such as 3DEM and serial femtosecond X-ray crystallography using X-ray free electron lasers (XFEL), and entirely new approaches to structure determination, such as integrative/hybrid methods (I/HM) (33), are coming to the fore. These advances will require major additions to the PDBx/mmCIF data dictionary and changes in the OneDep system to properly represent the outcomes of multi-scale/time course structure determinations and to capture structural information, experimental data and metadata. The wwPDB has begun working with community experts in XFEL and I/HM to develop PDBx/mmCIF dictionary extensions for data standards that can be used in the OneDep system to support these techniques.
2. Scaling up the day-to-day operations: These accelerating changes in the science and technology of structural biology will also present challenges for the *Biocurators*; e.g. XFEL and I/HM domain expertise will be required. Both the OneDep system and biocuration practices need to evolve in the face of these changes. As the number of depositions per year increases and the size and complexity of incoming structures grows, there is a pressing need for further automation of the wwPDB biocuration processes. Moreover, with growing concerns about accuracy and reproducibility across the sciences, the OneDep validation module will require further enhancement.
3. Training and retention of workforce: The wwPDB places considerable emphasis on training and retention of our highly skilled *Biocurators*. We are committed to ensuring that biocuration is a rewarding and valued career within our organization. Looking more broadly across biology and medicine, the scientific community depends critically on ready access to comprehensive, high-quality primary archival data resources. The International Society for Biocuration (www.biocuration.org/) helps *Biocurators* develop throughout their professional careers through annual International Biocuration Conferences, workshops, communication forums, etc.

In conclusion, we wish to reiterate that the scientific community, and society in general, requires a durable and permanent record of the results of research. For these data to be *Findable, Accessible, Interoperable* and *Reusable*, they must be expertly and thoroughly curated. Ideally, experimental data and metadata should be prepared for archiving prior to publication, not after the fact (or never as is unfortunately often the case). Since its inception in 1971, the PDB has served as the exemplar of a first-rate curated scientific data archive. Skilled *Biocurators*, enabled with stringent software checks, apply their domain expertise to ensure access to high-quality data for *Data Depositors* and *Data Consumers* alike. Since 2003, the global wwPDB partnership has provided a robust framework for expert biocuration in furtherance of its mission to maintain and grow a sustainable archive of structural biology data made freely available without limitations on data usage for researchers, educators, students and the curious public around the globe.

## Usage notes

PDB data are public and open access (ftp://ftp.wwpdb.org/pub/pdb/data/structures/) for experts and non-experts with no limitation on usage. We ask users to cite ‘Berman *et al*. (2)’ when PDB data are referenced. The PDBx/mmCIF data dictionary and CCD are defined at mmcif.wwpdb.org/ and www.wwpdb.org/data/ccd, respectively. Information about the OneDep system including tutorials and an FAQ list is available at www.wwpdb.org/deposition/system-information. The documentation for wwPDB biocuration procedures and policies is maintained at www.wwpdb.org/documentation/annotation.

## Funding

This work was supported by National Science Foundation; National Institutes of Health and Department of Energy (grant number DBI-1338415) at Research Collaboratory for Structural Bioinformatics (RCSB) Protein Data Bank (PDB); by European Molecular Biology Laboratory-European Bioinformatics Institute; Wellcome Trust (grant number 75968, 88944 and 104948); Biotechnology and Biological Sciences Research Council (grant number BB/G022577/1, BB/J007471/1, BB/K016970/1, BB/K020013/1, BB/M013146/1, BB/M011674/1, BB/M020347/1 and BB/M020428/1); European Union (grant number 284209 and 675858); Medical Research Council (grant number MR/L007835/1) at PDB in Europe (PDBe); by Japan Science and Technology—National Bioscience Database Center (grant number 17933363) at PDB Japan (PDBj) and PDBj-Biological Magnetic Resonance Bank (BMRB); by National Institute of General Medical Sciences (grant number GM109046) at BMRB and by National Institute of General Medical Sciences (grant number GM079429) at EMDataBank partner sites at PDBe and RCSB PDB.
